# Long‐term and interactive effects of different mammalian consumers on growth, survival, and recruitment of dominant tree species

**DOI:** 10.1002/ece3.6578

**Published:** 2020-07-20

**Authors:** J. Hall Cushman, Laura E. Saunders, Tyler K. Refsland

**Affiliations:** ^1^ Department of Natural Resources & Environmental Science University of Nevada Reno NV USA; ^2^ Department of Biology Sonoma State University Rohnert Park CA USA

**Keywords:** context‐dependent effects, co‐occurring consumers, individual and interactive effects, large and small mammals, long‐term experiments, oak woodlands, plant–herbivore interactions, tree decline, tree recruitment and regeneration

## Abstract

Throughout the world, numerous tree species are reported to be in decline, either due to increased mortality of established trees or reduced recruitment. The situation appears especially acute for oaks, which are dominant features of many landscapes in the northern hemisphere. Although numerous factors have been hypothesized to explain reductions in tree performance, vertebrate herbivores and granivores may serve as important drivers of these changes. Here, using data from 8‐ and 14‐year‐old exclosure experiments, we evaluated the individual and interactive effects of large and small mammalian herbivores on the performance of three widespread oak species in California—coast live oak (*Quercus agrifolia*), California black oak (*Q. kelloggii*), and Oregon white oak (*Q. garryana*). Although impacts varied somewhat by species and experiment, herbivory by black‐tailed deer (*Odocoileus hemionus columbianus*) reduced the height and survival of juvenile coast live oaks and altered their architecture, as well as reduced the abundance of black oak seedlings, the richness of woody species and the cover of nonoak woody species. Small mammals (*Microtus californicus* and *Peromyscus maniculatus*) had even more widespread effects, reducing the abundance of black oak seedlings and the height and cover of all three oak species. We also detected numerous interactions between small mammals and deer, with one herbivore having positive or negative effects on oak abundance and cover when the other herbivore was either present or absent. For example, deer often had negative effects on seedling abundance only when, or even more so when, small mammals were present. In summary, mammalian consumers play crucial roles in limiting oak recruitment by reducing seedling abundance, maintaining trees in stunted states, and preventing them from reaching sapling stages and becoming reproductive. Interactions between large and small mammals can also alter the intensity and direction of their effects on trees.

## Graphical abstract

Our research highlights the importance of mammalian consumers in mediating the recruitment of dominant trees species that are hypothesized to be in decline. It reveals that large and small mammals have strong individual and interactive effects that greatly limit key processes associated with forest regeneration.

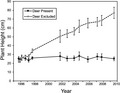

## 1 INTRODUCTION

Throughout the world, an increasing number of trees species are reported to be declining in abundance (Allen et al., 2010; Cohen et al., 2016; van Mantgem et al., 2009; Millar & Stephenson, 2015; Trumbore, Brando, & Hartmann, 2015). In many cases, this decline is due to increased mortality of established trees, whereas in other cases it involves reduced recruitment, with younger trees being either less abundant or failing to reach the adult stage (Duwyn & MacDougall, 2015; Fei, Kong, Steiner, Moser, & Steiner, 2011; Gibbons et al., 2008; Kueppers, Snyder, Sloan, Zavaleta, & Fulfrost, 2005; Manning, Fischer, & Lindenmayer, 2006). Understanding the patterns of tree decline and their drivers is of great importance given that forests, woodlands, and savannas cover 30% of the world's terrestrial landscapes (FAO, 2006) and have major influences on ecological systems, including carbon sequestration, water purification, and habitat provisioning (Trumbore et al., 2015).

Although many factors have been implicated as drivers of tree decline—either acting individually or in combination—mammalian consumers are increasingly recognized as factors that can reduce tree recruitment in many forests of the world (Bradshaw & Waller, 2016; Davis, Tyler, & Mahall, 2011; Faison, DeStefano, Foster, Rapp, & Compton, 2016; Long, Pendergast, & Carson, 2007). Due to intensifying human land use and declines in large carnivore populations, many ungulates have expanded their ranges and/or increased in abundance worldwide in recent decades, and are frequently implicated in the reduced performance and abundance of dominant trees in many forested ecosystems (Beschta & Ripple, 2009; Martin, Arcese, & Scheerder, 2011; Ripple & Beschta, 2007b; Russell, Zippin, & Fowler, 2001; Takatsuki, 2009; Tanentzap et al., 2011). Rodents and other small mammals have also been found to have strong effects on recruitment of trees and other woody plant species (López‐Sánchez et al., 2019; MacDougall, Duwyn, & Jones, 2010; McLaughlin & Zavaleta, 2013; Tyler, Davis, & Mahall, 2008).

Although multiple mammalian consumer taxa commonly co‐occur in the same system, studies typically address the effects of either only a single consumer species on focal plant populations and communities or all consumer species together (Faison et al., 2016). Given the practical constraints of conducting large‐scale experiments in the field, focusing on individual species is understandable. However, such approaches likely result in incomplete and perhaps even misleading conclusions, given that one consumer species may impact a system in ways that alter the effect that other co‐occurring consumer species have on the same host plants and/or community (Ellis & Cushman, 2018; Maclean, Goheen, Doak, Palmer, & Young, 2011; Parsons, Maron, & Martin, 2013; Pringle, Young, Rubenstein, & McCauley, 2007). In addition to the simple additive effect in which multiple consumer species may feed on the same host plant species, thus increasing their total impact on individual plants, several nonadditive effects may also emerge: (a) Multiple consumers may feed on different plant species and alter the competitive interactions between these hosts; (b) one consumer species may alter the availability of a shared host plant to another consumer; and/or (c) one consumer species may modify prevailing ecological conditions such that the feeding behavior or intensity of a second consumer is altered. For example, Muñoz, Bonal, and Díaz (2009) showed that wild ungulates (red deer [*Cervus elaphus*], roe deer [*Capreolus capreolus*], and boars [*Sus scrofa*]) reduced plant species richness and shrub cover, which in turn caused small mammals to alter their seed‐caching behavior in ways that reduced the recruitment of oak seedlings. Studies such as this indicate that the effects of one consumer species must often be understood in the context of other consumer taxa in the system. Indeed, Ritchie and Olff (1999) stated that multiple consumer species can have either compensatory or additive effects on the plant community, depending on the particular factors that limit plant species in a given system, and concluded that more field research was required to assess the impacts of multiple consumer on plant populations and communities. However, two decades after this paper was published, we are aware of only a handful of studies (Alberti, Bakker, van Klink, Olff, & Smit, 2017; Bakker, Ritchie, Olff, Milchunas, & Knops, 2006; Davidson et al., 2010; Howe & Brown, 2000; Huntzinger, Karban, & Cushman, 2008; Maclean et al., 2011; Wilson, Ruscoe, Burrows, McElrea, & Choquenot, 2006) that have conducted such factorial assessments to elucidate the impacts of different mammalian consumer species on plant populations.

It is particularly urgent to understand the impacts of multiple mammalian consumers on oaks because these trees are thought to be declining in many areas (Abrams, 2003; López‐Sánchez et al., 2019; López‐Sánchez, Perea, Dirzo, & Roig, 2016; MacDougall et al., 2010; Tyler et al., 2008; Tyler, Kuhn, & Davis, 2006; Zavaleta, Hulvey, & Fulfrost, 2007). Oaks are a dominant group throughout much of the temperate, tropical, and subtropical northern hemisphere and serve as crucial food sources for many wildlife species (McShea et al., 2007). Researchers in many locations across Europe, Asia, Central America, and North America have found that oak recruitment and stand structure are not adequate to sustain forests and other oak‐defined habitats (Abrams, 2003; MacDougall et al., 2010; Ripple & Beschta, 2007a, 2008; Tyler et al., 2006; Zavaleta et al., 2007). This situation is especially striking in California where natural recruitment is thought to be low for numerous oak species, and there is mounting concern that regeneration may be insufficient to maintain existing stands (Tyler et al., 2006, see review by Tyler et al. (2008)). Unfortunately, the existence, extent, and causes of these apparent recruitment reductions are often unclear (Zavaleta et al., 2007, although see Ripple & Beschta, 2008). In addition, as noted by Tyler et al. (2006), the majority of studies evaluating the population dynamics of oaks in California have been of short duration and focused on factors controlling acorn and seedling recruitment. Although these studies have provided valuable insights about important life‐history stages, a comprehensive understanding of oak recruitment requires that we also conduct field studies of longer duration and expand our focus to include factors that influence the transition from seedlings to saplings.

Here, we summarize research from two long‐term exclosure experiments in northern California that address the individual and interactive effects of large and small mammalian consumers on oak performance and the surrounding understory community. First, we use a 14‐year‐old exclosure experiment to assess the impact of herbivory by black‐tailed deer (*Odocoileus hemionus columbianus*) on the growth, architecture, and survival of juvenile coast live oak (*Quercus agrifolia*), an abundant tree species in central California (Barrett, Gatziolis, Fried, & Waddell, 2006). Our research sought to explore the role of deer herbivory in mediating the transition from the juvenile to sapling stage because, like many areas, our site had an abundance of seedlings and established reproductive trees but a near‐complete absence of sapling‐stage individuals. Second, we use an eight‐year‐old factorial exclosure experiment to evaluate the individual and interactive effects of black‐tailed deer and small mammals (primarily meadow voles, *Microtus californicus*, and deer mice, *Peromyscus maniculatus*) on the growth and abundance of seedlings and juveniles for three dominant trees: coast live oak, Oregon white oak (*Q. garryana*), and California black oak (*Q. kelloggii*). We also consider how these factorial manipulations affect the cover of understory oak species, nonoak woody species, woody species richness, and herbaceous plant biomass. Such data on nonoak vegetation can help identify indirect pathways by which mammalian consumers influence oaks. Collectively, results from these two long‐term experiments will provide insight about the role of mammalian consumers in mediating the performance of dominant oak species and their regeneration during a period of suspected tree decline.

## 2 METHODS

### 2.1 Study system

We conducted this study at two sites in northern California: Sonoma State University's Fairfield Osborn Preserve (FOP; 38°34ʹN, 122°59ʹW) and Stanford University's Jasper Ridge Biological Preserve (JRBP; 37°40ʹN, 122°23ʹW). Both sites are characterized by a Mediterranean‐type climate, with moderate rainy winters and cool foggy summers with little or no precipitation. FOP is 170 ha in size and located on the western slope of Sonoma Mountain in eastern Sonoma County, 50 km north of San Francisco. JRBP is 480 ha in size and located in the eastern foothills of the Santa Cruz Mountains in San Mateo County, 30 km southeast of San Francisco. Our study plots at these sites occurred at 500 and 100 m elevation, respectively.

Both research sites are located in oak woodlands or savannas that support abundant populations of native oaks. Coast live oak, Oregon white oak, and California black oak were abundant at the FOP study area, whereas mostly coast live oak occurred at the JRBP study area. All three species grow to 20–40 m in height, live 200–400 years, and produce copious acorn crops. Coast live oak is evergreen whereas black and white oak are deciduous. Larger mature trees were not rooted in our plots but overhung them and formed a canopy. The forest canopy at both sites was composed primarily of coast live oak, California black oak, and Oregon white oak, with a small amount of bay laurel (*Umbellularia californica*). Nonoak juvenile trees that grew in our plots during the course of the experiments included buckeye (*Aesculus californica*), bay laurel, California black walnut (*Juglans californica)*, tanoak (*Lithocarpus densiflorus*), Douglas fir (*Pseudotsuga menzeisii*), and toyon (*Heteromeles arbutifolia*). Nontree woody species encountered in our plots included poison oak (*Toxicodendron diversilobum*), honeysuckle (*Lonicera hispidula*), snowberry (*Symphoricarpos albus),* California blackberry (*Rubus californicus*), Himalayan blackberry (*Rubus discolor*), and thimbleberry (*Rubus parviflorus*). The herbaceous understory was composed of a mixture of exotic and native grasses and forbs.

Both sites had abundant populations of black‐tailed deer (*Odocoileus hemionus columbianus*), meadow voles (*Microtus californicus*), and deer mice (*Peromyscus maniculatus*). All three taxa are well known to feed on the foliage, terminal and lateral buds and acorns of oaks (Bartolome et al., 2002; Griffin, 1971; Jameson & Peeters, 2004; MacDougall et al., 2010; Tyler et al., 2006, 2008; Weckerly, 1994). Pocket gophers (*Thomomys bottae*) are known to feed on acorns and oak seedlings (Jameson & Peeters, 2004) but were not common where our exclosure experiments were conducted. Similarly, California ground squirrels (*Otospermophilus beecheyi*) are known to feed on acorns but were not especially common at our study sites.

### 2.2 Experiment 1: Deer exclosure experiment

#### 2.2.1 Experimental design

In September 1996, we established an exclosure experiment in oak woodland/savanna habitat at FOP to quantify the long‐term impacts of herbivory by black‐tailed deer on the growth, architecture, and survival of juvenile coast live oak. This 14‐year‐old experiment involved 50 juvenile oak trees that, at the start of the study, ranged from 15 to 53 cm in height. Although small in size aboveground, these individuals were well past the seedling stage (i.e., they were no longer attached to acorns) and possessed well‐developed root crowns immediately below the soil surface but were not reproductive. Juvenile oaks had a shrubby architecture without a clear main stem, suggesting they have commonly resprouted after past herbivore damage (Griffin, 1971). We grouped these oaks in pairs (*n* = 25), with individuals in pairs located within 2 m of each other and matched for similarity in size, architecture, topography, and microhabitat. We randomly assigned one member of each pair to receive deer fencing whereas the other was left unmanipulated and served as a control. Deer exclosures consisted of cylindrical mesh fencing surrounding each tree that was 1.25 m tall and secured to the ground with multiple steel U‐shaped stakes. The diameter of each cylindrical fence was determined by the size of the juvenile oak, but typically was 50 cm. In a number of instances, cylindrical fences had to be enlarged to accommodate the growth of juvenile oaks during this 14‐year‐long experiment. These metal fences effectively prevented deer from feeding on the 25 manipulated oaks for the duration of this experiment (J. H. Cushman, personal observation). In addition, small mammals (meadow voles, deer mice, and ground squirrels) were not excluded by the deer fencing (J. H. Cushman, personal observation).

#### 2.2.2 Oak performance and survival

On 14 occasions during a 14‐year period (September 1996, November 1996, February 1997, September 1997, February 1998, September 1998, September 2002, September 2003, September 2004, June 2005, September 2006, January 2008, September 2008, and June 2010), we assessed the status and height of all 50 juvenile coast live oaks included in the experiment. In addition, in year 12 of the experiment (February 2008), we determined for each surviving individual the number of basal stems (juvenile trees regularly had multiple basal stems emerging from a single root crown), cumulative basal stem diameter, total number of leaves, and the fraction of these that were damaged by insect herbivores. In July 2010, we also quantified the maximum diameter of each tree's canopy and the diameter perpendicular to the midpoint of this measurement. We then used these two values to estimate canopy area of each tree with the formula for an ellipse (π[maximum diameter × perpendicular diameter]/4). To evaluate the influence of herbivory on tree shape, we also calculated the ratio of height to canopy diameter.

### 2.3 Experiment 2: Deer and small‐mammal exclosure experiment

#### 2.3.1 Experimental design

In December 2000, we established a second consumer‐exclosure experiment at FOP and JRBP to evaluate the influence of black‐tailed deer and small mammals (meadow voles and deer mice) on the abundance, survival, and growth of oak seedlings, defined as young individuals with acorns still attached or only recently detached, and juveniles, defined as prereproductive individuals less than 1‐m tall. We established 16 plots (6 × 6 m) at each of the two preserves. To control for underlying spatial variation, we grouped the plots at each preserve into four blocks, with all blocks containing one plot per treatment combination (*n* = 4 per treatment per site). For each block, we located plots within 1–3 m of each other, randomly assigned treatment combinations, and matched plots for topography, understory woody composition, and percent canopy cover of overstory trees. Although all three oak species were present at both study sites, our plots at FOP contained predominately white oak and black oak, whereas JRBP contained predominately coast live oak. We randomly assigned each plot within a block to receive one of the following four manipulations: Small mammals excluded, deer excluded, both types of mammals excluded, and neither manipulated (control). All plots had metal t‐posts at their corners, with post heights being either 1 m (controls and small‐mammal exclosures) or 2.25 m (deer and deer/small mammal exclosures). Deer‐exclusion plots had 2‐m high woven‐wire fencing that extended to within 0.4 m of the ground, whereas small mammal exclosures had 0.6‐m high woven‐wire fencing/hard cloth with a band of flashing at the top (this fencing was buried approximately 60 cm underground). Both types of fencing were used to jointly exclude/reduce deer and small mammals. During this 8‐year‐long experiment, we never observed deer or their dung inside plots with the tall fencing. In addition, using a combination of direct observation and dung counts, we determined that the low fencing did not deter deer from using the small‐mammal exclosures. Small mammals are notoriously difficult to exclude from areas, so we tested the efficacy of our exclosures using a combination of small‐mammal trapping and smoked aluminum track plates at both sites. In 2000, we estimated small‐mammal populations using Sherman live traps for 10 nights. In 2008, we again performed live trapping in all plots for three nights and track‐plate sampling for seven nights. In both years, our sampling consistently revealed that the lower fences greatly reduced the abundance of small mammals in plots but did not exclude them entirely (J. H. Cushman and L. E. Saunders, unpublished results). Similar to Experiment 1, California ground squirrels were not affected by either type of fencing.

#### 2.3.2 Oak abundance and height

In May 2001, five months after the exclosures were established, we found there were no treatment effects on oak seedling abundance or the percent cover of juvenile oaks and nonoak woody species (J. H. Cushman and L. E. Saunders, unpublished data). In May and June 2008, eight years after the consumer‐exclosure experiment was established, we assessed the effects of deer and small mammals on the abundance and size of young oaks. Although oak acorn production and recruitment are well known to be highly variable among years (Koenig, Mumme, Carmen, & Stanback, 1994), our single measurement of oak seedling and juvenile abundance was intended to quantify the long‐term, cumulative effects of herbivores on recruitment rather than characterize year‐to‐year variability. Excluding a 50‐cm border around the perimeter of each plot to minimize edge effects, we determined the abundance of oaks in all plots and recorded the species, main stem height and number of leaves of each individual. We then categorized each oak in all plots as being either a seedling or juvenile as follows. Individuals categorized as seedlings were less than 15 cm in height and lacked a well‐developed root crown (in many cases, individuals also had acorns still attached). Individuals categorized as juveniles were greater than or equal to 15 cm in height and possessed a well‐developed root crown just below the soil surface. These latter individuals were clearly prereproductive but had not yet become saplings.

#### 2.3.3 Woody species richness and cover

From late April through June 2008, we sampled woody vegetation in our 32 plots to assess how mammalian consumers influenced woody plant species richness and cover. We performed whole‐plot surveys to determine the woody species present in each plot (both oaks and nonoaks). To estimate percent cover, we recorded all woody species encountered at 100 points distributed equally among 10 transects per plot. We stratified transects evenly across each plot at 60‐cm intervals, creating a 10 × 10 grid. Excluding a 50‐cm strip around the perimeter of each plot to minimize edge effects, each transect was 5‐m long and we sampled points every 50 cm along each transect. Because forest canopy cover is known to have significant effects on oak seedling survival, growth, and abundance in other systems (Beckage & Clark, 2005; Borchert, Davis, Michaelsen, & Oyler, 1989), we estimated forest canopy cover using a densiometer at the same 100 points used to assess woody cover.

#### 2.3.4 Herbaceous plant biomass

To assess whether large and small herbivores altered herbaceous plant biomass, we harvested all aboveground herbaceous biomass, living or dead, to the surface of the leaf litter from within five 110 cm^2^ quadrats per plot during peak biomass in June 2008. Biomass was pooled by plot and oven‐dried at 60°C for 48 hr.

### 2.4 Statistical analyses

#### 2.4.1 Experiment 1

To assess the influence of deer herbivory on the height of juvenile coast live oaks through time, we used a linear mixed model in the “nlme” package (Pinheiro, Bates, DebRoy, & Sarkar, 2020) in R (R Development Core Team, 2019). To account for temporal autocorrelation, we used a first‐order autoregressive covariance structure within the linear mixed model. Log‐transformed juvenile coast live oak height was modeled as a function of deer treatment (present, excluded), year and their interaction. A random intercept and slope were specified such that the intercept and effect of year on height were allowed to vary by tree nested within pair (1–25).

To evaluate the impact of deer herbivory on the various growth measurements obtained in 2008 or 2010, we performed a series of mixed models with deer treatment (present, excluded) as the fixed effect and pair (1–25) as a random intercept. We used linear mixed models to analyze the effect of deer herbivory on cumulative basal stem diameter, canopy area, canopy height‐to‐width ratio, and the fraction of leaves damaged by insect herbivores. We log‐transformed data whenever necessary to normalize them and equalize variances. We used negative binomial generalized linear mixed models (GLMMs) to evaluate whether the number of basal stems and total number of leaves were affected by deer treatment. To test whether the survival of juvenile oaks after 14 years was independent of the deer treatment (present, excluded), we performed a logistic regression and assessed the overall effect of deer treatment with a Wald chi‐square test using the “aod” package in R (Lesnoff & Lancelot, 2012).

#### 2.4.2 Experiment 2

We used negative binomial GLMMs to examine the influence of mammalian consumers on understory oak abundance, with small mammal treatment (present, reduced), deer treatment (present, excluded) and their interaction as fixed effects and block (1–4) as a random effect. To evaluate the influence of mammalian consumers on understory oak height, percent cover of understory oak and nonoak woody species as well as herbaceous biomass, we performed a series of linear mixed models in R, with the same fixed effect terms and interactions as in the GLMMs. For the linear mixed models, we log‐transformed all data when they were non‐normal or exhibited unequal variances. When analyzing oak abundance and height, we ran separate models for each site (FOP, JRBP) and size class (seedling, juvenile) because only black and white oak seedlings were present at FOP while only coast live oak seedlings and juveniles were present at JRBP. We included both sites in models for percent understory oak cover, percent cover of nonoak woody species, woody species richness, and herbaceous biomass because results did not change significantly when sites were modeled separately. Percent canopy cover of overstory trees was not a significant predictor variable for any of our response variables; therefore, we dropped it from all models. All analyses were completed in R using the “glmmTMB” package (Brooks et al., 2017) for the GLMM models and the “nlme” package (Pinheiro et al., 2020) for the linear mixed models. Significant treatment effects were evaluated at *α* = 0.05, and trends (i.e., marginal effects) were evaluated at *α* = 0.10.

## 3 RESULTS

### 3.1 Experiment 1: Deer exclosure experiment

**FIGURE 1:**
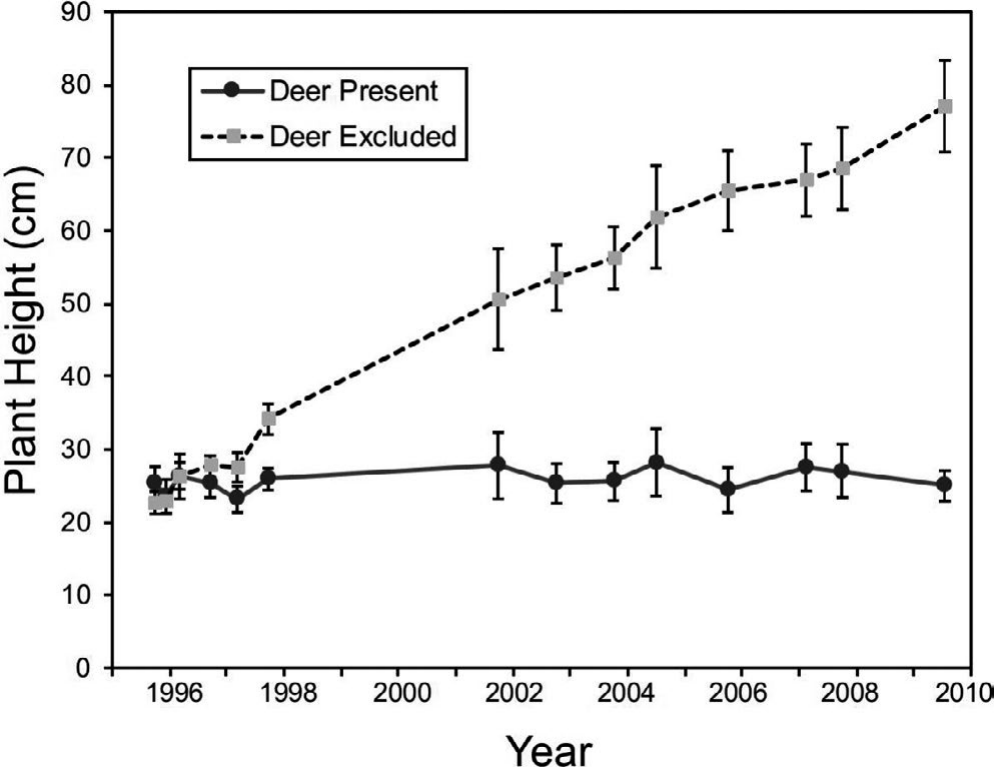
Mean (±1 *SE*) height of juvenile coast live oak over a 14‐year period as a function of the presence or absence of black‐tailed deer

Results from a linear mixed model with repeated‐measures indicated that deer had a significant effect on the height of juvenile coast live oaks (*F*_1,24_ = 163.29, *p* < .0001), with their influence increasing through time (treatment × year interaction: *F*_1,417_ = 96.86, *p* < .0001). After 14 years, deer herbivory caused a 3.1‐fold reduction in the height of oaks, with the height of oaks exposed to deer remaining virtually unchanged over the study period (Figure 1).

**FIGURE 2:**
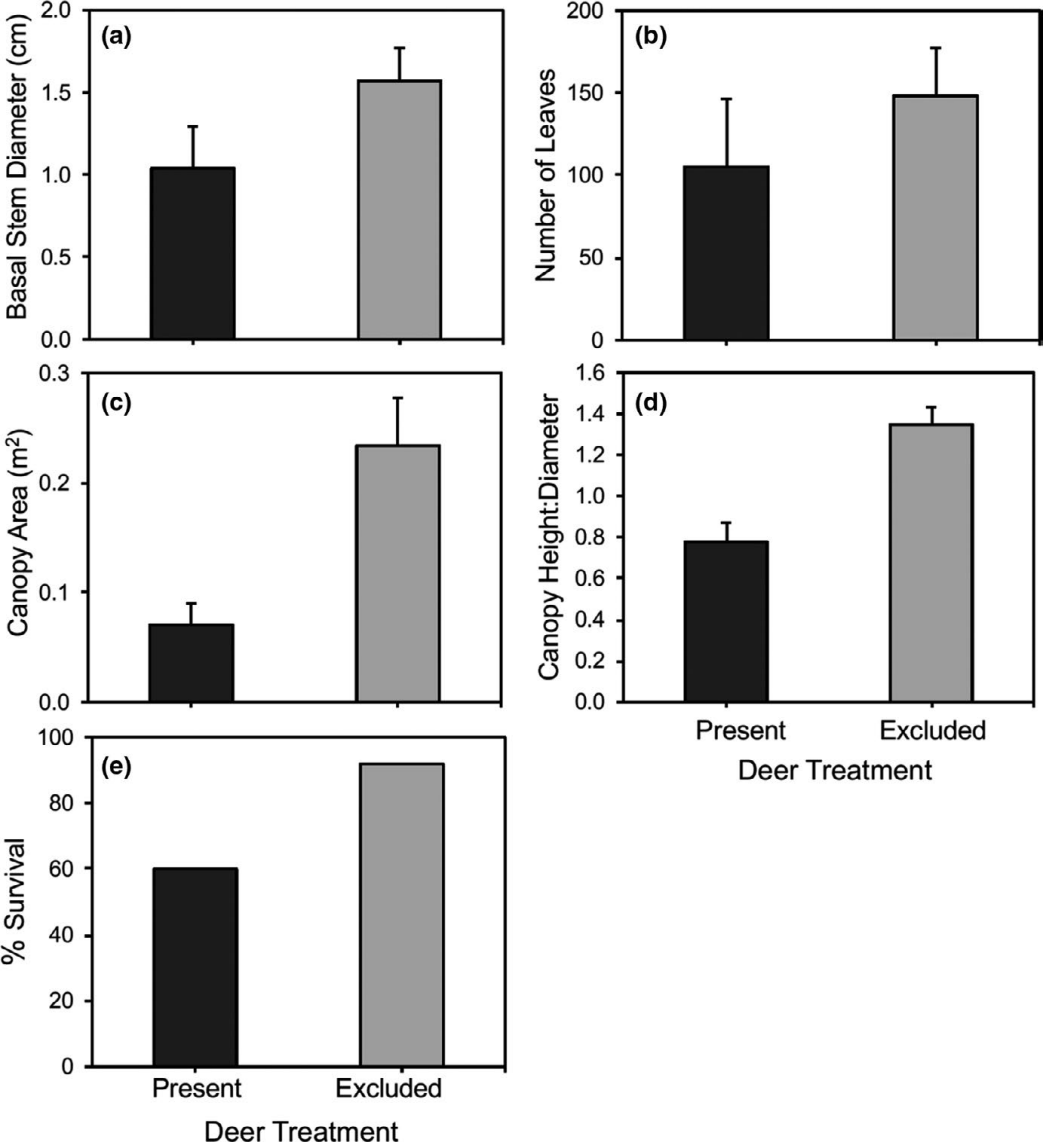
Mean (±1 *SE*) performance and percent survival of juvenile coast live oak as a function of the presence or absence of black‐tailed deer. Data show the effects of these herbivores after 12 (a, b) and 14 years (c, d, e)

After 12 years, linear mixed models indicated that deer caused a 1.5‐fold reduction in basal stem diameter (*F*_1,18_ = 15.02, *p* = .0011; Figure 2a) and a 1.4‐fold reduction in the number of leaves per plant (*β* = −0.51, *p* = .009; Figure 2b). After 14 years, deer caused a 3.3‐fold reduction in canopy area (*F*_1,14_ = 34.17, *p* < .0001; Figure 2c) and a significant change in the general shape of trees, with browsing converting oaks from tall, slender individuals to short, wide ones (*F*_1,14_ = 27.88, *p* < .0001; Figure 2d). In addition, a logistic regression revealed that the survival of juvenile oaks was affected by deer (*χ* = 5.8, *df* = 1, *p* = .016), with trees protected from deer exhibiting 35% greater survival than those exposed to it (Figure 2e). In contrast, deer did not have a significant effect on the number of basal stems per tree (deer present mean = 2.55 ± 0.46 vs. deer excluded mean = 2.30 ± 0.18; *p* = .61) or the percent of leaves with insect damage (deer present mean = 70.36 ± 4.16 vs. deer excluded mean = 78.24 ± 3.28; *F*_1,18_ = 2.37, *p* = .14).

### 3.2 Experiment 2: Deer and small mammal exclosure experiment

**FIGURE 3:**
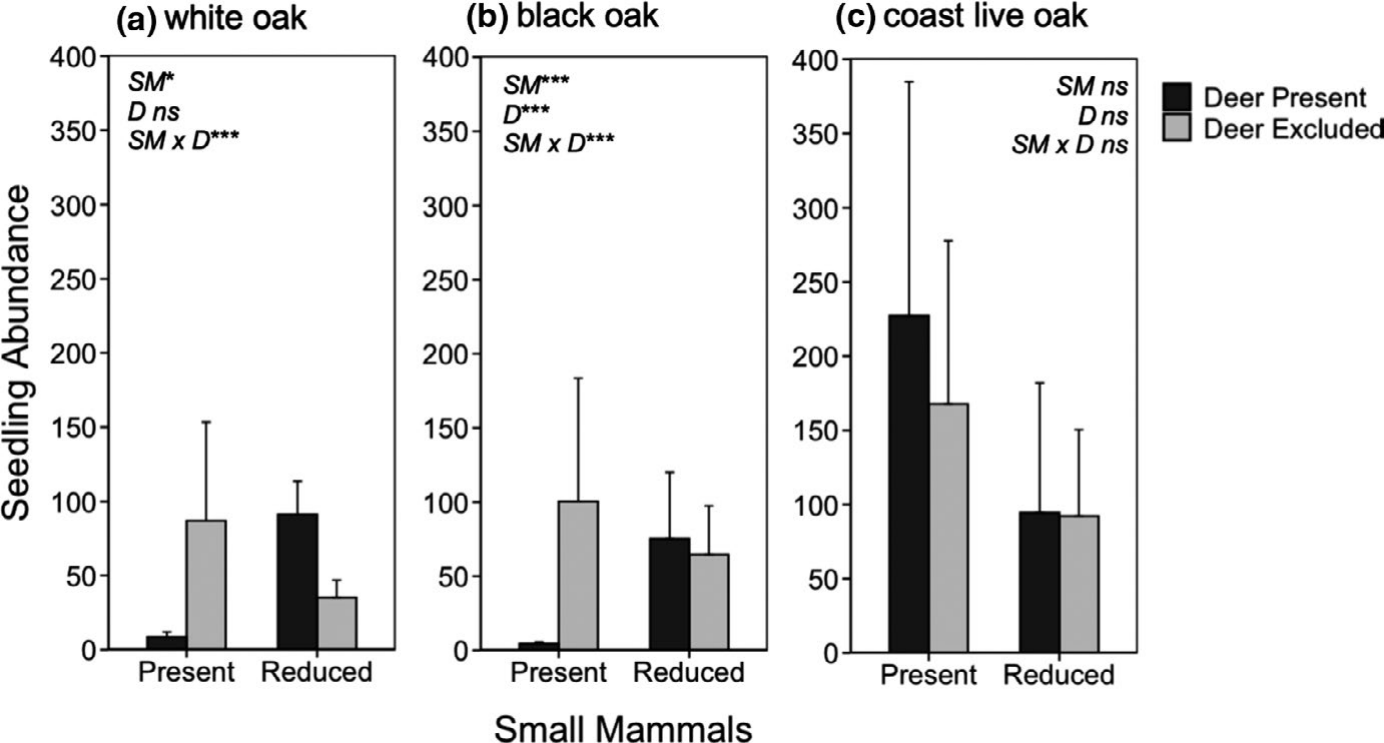
Mean (±1 *SE*) seedling abundance for white oak (a), black oak (b), and coast live oak (c) as a function of whether deer and small mammals were present or excluded/reduced. Data show seedling abundance in 2008, 8 years since treatment initiation. White and black oak seedlings were found at FOP, whereas only coast live oak seedlings were found at JRBP. Asterisks indicate level of significance: *p < .05; **p < .01; ***p < .001

Negative binomial GLMMs revealed that on their own, both deer and small mammals significantly reduced the abundance of California black oak seedlings (*β* = −2.32, *p* < .001; *β* = −2.15, *p* < .001; respectively; Figure 3b). For Oregon white oak seedlings, deer did not influence abundance (*p* = .15) whereas small mammals significantly reduced their abundance (*β* = −1.58, *p* = .012; Figure 3a). For seedling abundance of both black and white oaks, there was a significant deer × small mammal interaction term (*β* = 2.56, *p* < .001; *β* = 2.07, *p* < .001; respectively). As shown in Figure 3, small mammals had negative effects on seedling abundance of both white and black oaks when deer were present, and for white oak only, small mammals had a positive effect when deer were absent. In contrast, neither deer, small mammals, nor their interaction affected the seedling abundance of coast live oak (*p* = .68; *p* = .11; *p* = .47; respectively; Figure 3c). Although deer and small mammals did not on their own influence the abundance of juvenile coast live oaks (*p* = .10; *p* = .16; respectively), there was a significant deer × small mammal interaction (*β* = 1.19, *p* = .047; Figure 5a), with the effects of each species being significant when the other species was present.

**FIGURE 4:**
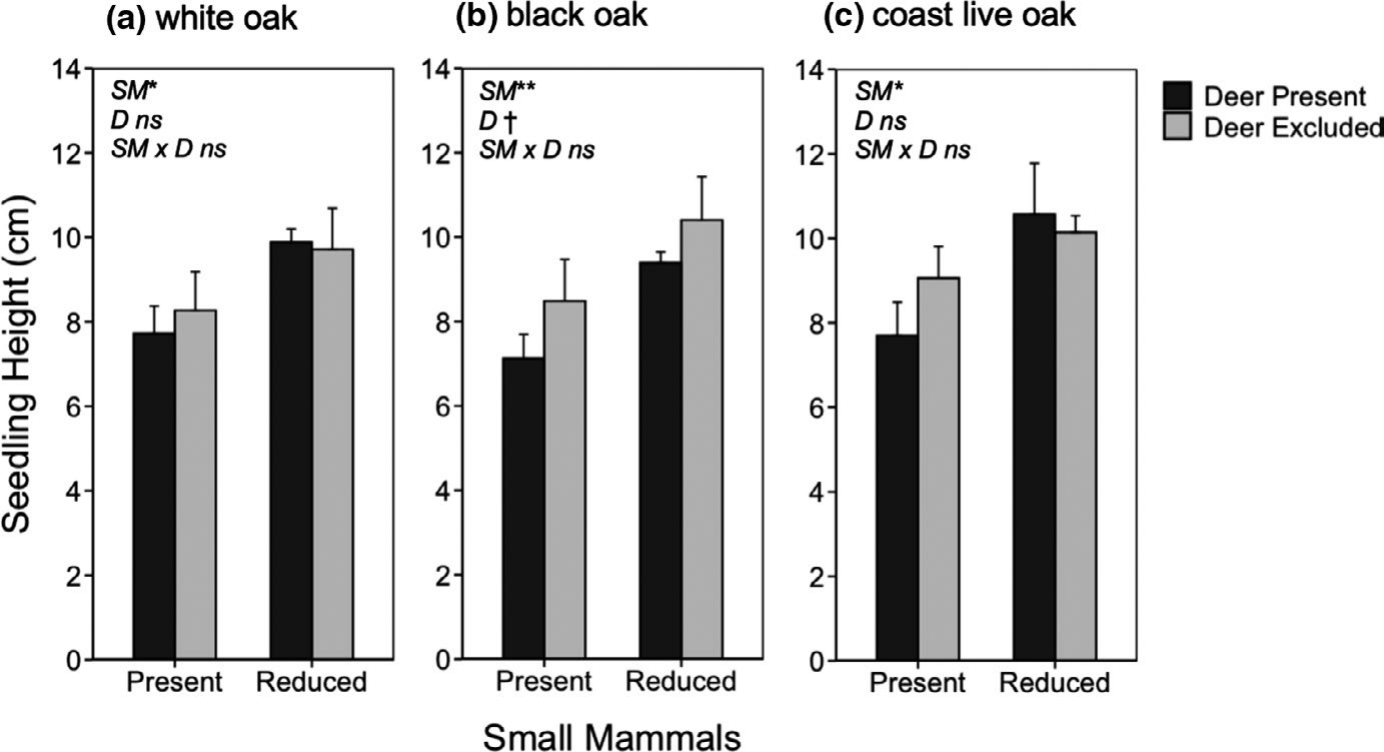
Mean (±1 *SE*) seedling height for white oak (a), black oak (b), and coast live oak (c) as a function of whether deer and small mammals were present or excluded/reduced. Data show seedling height in 2008, 8 years since treatment initiation. White and black oak seedlings were found at FOP, whereas only coast live oak seedlings were found at JRBP. Asterisks indicate level of significance: †*p* < .1; *p < .05; **p < .01; ***p < .001

**FIGURE 5:**
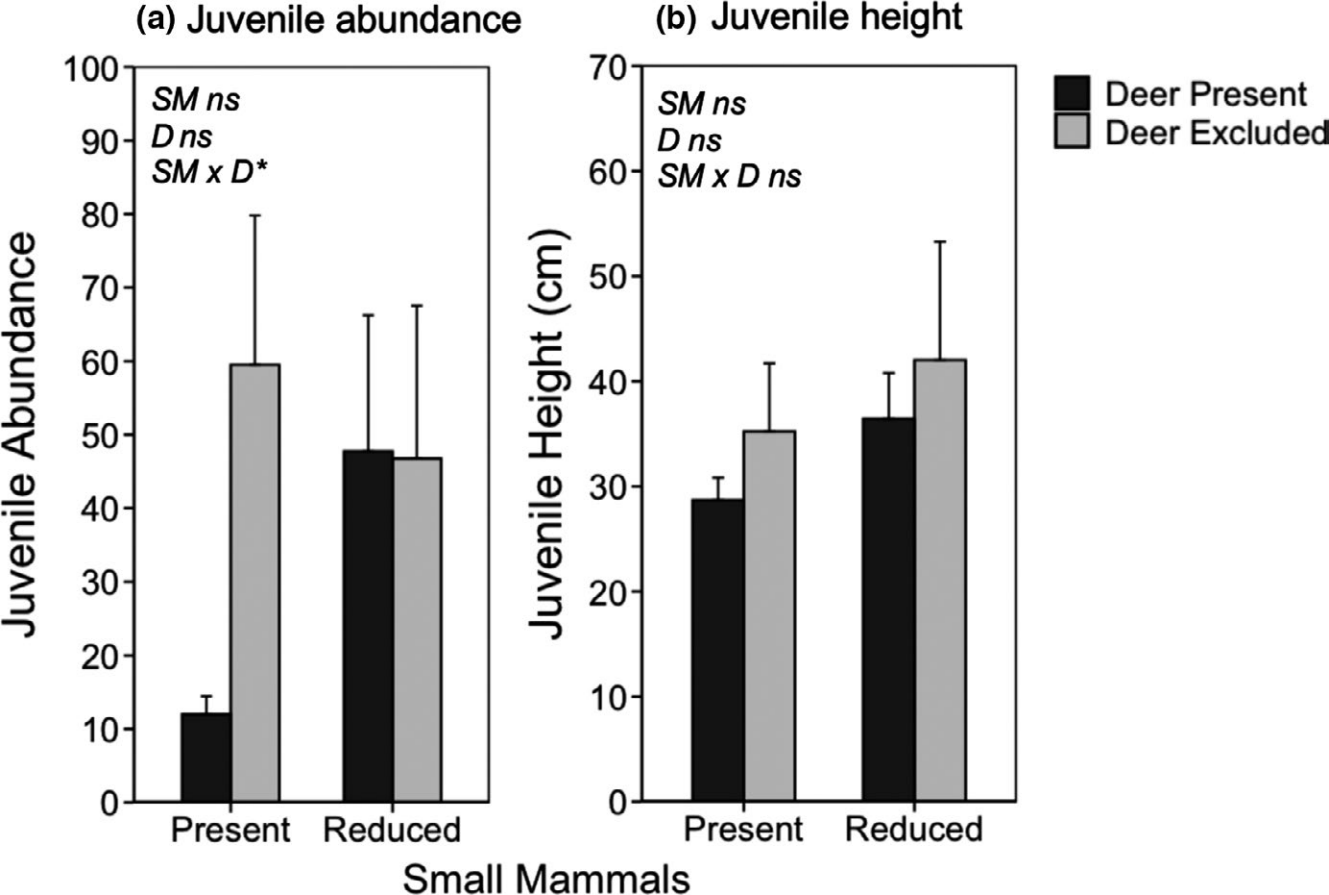
Mean (±1 *SE*) abundance (a) and height (b) of juvenile coast live oak as a function of whether deer and small mammals were present or excluded/reduced. Data show juvenile abundance and height in 2008, 8 years since treatment initiation. Asterisks indicate level of significance: †*p* < .1; *p < .05; **p < .01; ***p < .001

Linear mixed models indicated that small mammals significantly reduced seedling height for Oregon white, California black, and coast live oaks (*F*_1,9_ = 8.58, *p* = .0168; *F*_1,9_ = 16.14, *p* = .0030; *F*_1,9_ = 6.35, *p* = .0328; respectively). In contrast, deer did not have such effects on the height of white and coast live oak seedlings (*F*_1,9_ = 0.65, *p* = .442; *F*_1,9_ = 0.89, *p* = .326; respectively), although there was a trend for deer to reduce the height of black oak seedlings (*F*_1,9_ = 3.75, *p* = .085; Figure 4). The height of juvenile coast live oaks was not affected by small mammals or deer (*F*_1,9_ = 2.92, *p* = .121; *F*_1,9_ = 1.09, *p* = .323; respectively; Figure 5b). Furthermore, we did not detect significant deer × small mammal interactions for the height of white, black, and coast live oak seedlings (*F*_1,9_ = 0.04, *p* = .841; *F*_1,9_ = 0.35, *p* = .571; *F*_1,9_ = 0.72, *p* = .419; respectively) or the height of coast live oak juveniles (*F*_1,9_ = 0.25, *p* = .624).

**FIGURE 6:**
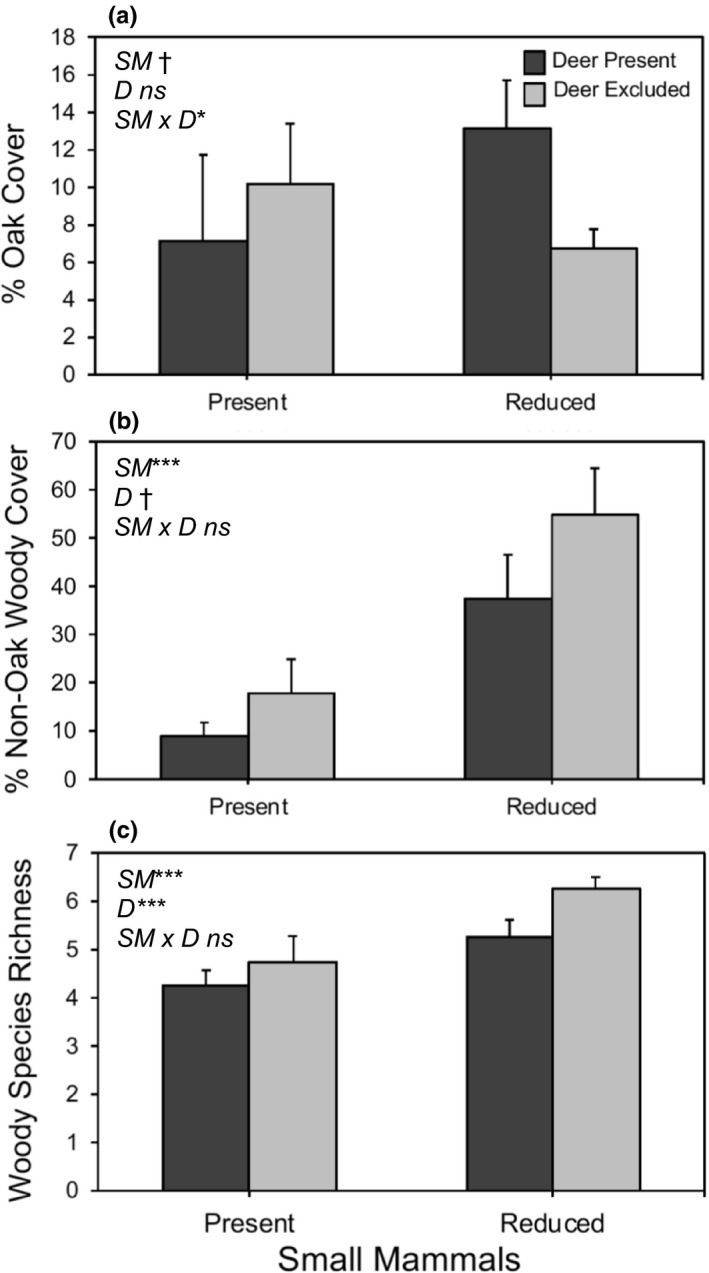
Mean (±1 *SE*) percent cover of understory oaks (a), nonoak woody species (b), and richness of woody species (c) as a function of whether deer and small mammals were present or excluded/reduced. Asterisks indicate level of significance: †*p* < .1; *p < .05; **p < .01; ***p < .001

By themselves, there was a trend for small mammals to negatively effect on understory oak cover (*F*_1,21_ = 3.79, *p* = .065) and deer had no effect at all (*F*_1,21_ = 0.04, *p* = .848). In contrast, there was a significant deer × small mammal interaction on understory oak cover (*F*_1,21_ = 5.49, *p* = .029). As shown in Figure 6a, deer had a negative effect on understory oak cover when small mammals were present but a positive effect when small mammals were absent. Small mammals significantly reduced the cover of nonoak woody understory species (*F*_1,20_ = 21.26, *p* = .0002; Figure 6b) and woody species richness (*F*_1,20_ = 28.26, *p* < .0001; Figure 6c). Deer reduced woody species richness (*F*_1,20_ = 15.9, *p* = .0007), and there was a trend for such effects with the cover of nonoak woody understory species (*F*_1,20_ = 3.52, *p* = .0747). In contrast, there was not a significant deer × small mammal interaction for either variable (*F*_1,20_ = 0.0, *p* = .99; *F*_1,20_ = 0.32, *p* = .578; respectively). In addition, herbaceous biomass was not affected by either deer (*F*_1,20_ = 0.82, *p* = .10) or small mammals (*F*_1,20_ = 2.04, *p* = .95), and there was not interaction between these two factors (*F*_1,20_ = 2.33, *p* = .14).

## 4 DISCUSSION

Our study highlights the importance of large and small mammalian consumers in driving long‐term patterns of recruitment for multiple oak species—as well as the cover and richness of understory oaks and nonoak woody species—and illustrates the frequent dependency of these effects on the interactions between different herbivore groups. Results from our 14‐year‐old deer exclosure experiment revealed that black‐tailed deer had strong negative effects on the growth and survival of juvenile coast live oaks as well as substantially altered their architecture. In addition, as summarized in Table 1, our eight‐year‐old exclosure experiment showed that small mammals and to a lesser extent deer each on their own commonly reduced the abundance, growth, cover, and/or richness of understory oaks and other woody species. However, this experiment also documented that interactions between small mammals and deer were often important as well, with the effect of one herbivore on oak abundance and cover changing when the other herbivore was either present or absent. Such context‐dependent outcomes of herbivores on woody species underscore the importance of conducting factorial experiments that allow for such complexities to emerge.

**TABLE 1:** Summary of results from an 8‐year‐old factorial exclosure experiment evaluating the effects of small mammals, deer and their interaction on a variety of plant response variables

Response variable	Model effects		
	Small mammals (SM)	Deer (D)	SM × D interaction
Abundance			
White oak seedlings	↓	No effect	↓When D & SM present
			↓When D & SM
			Excluded/reduced
Black oak seedlings	↓	↓	D when SM present
Coast live seedlings	No effect	No effect	no effect
Coast live juveniles	No effect	No effect	D when SM present
Height			
White oak seedlings	↓	No effect	No effect
Black oak seedlings	↓	↓[trend]	No effect
Coast live seedlings	↓	No effect	No effect
Coast live juveniles	No effect	No effect	No effect
Other			
% Oak cover	↓[trend]	No effects	D when SM reduced
% Nonoak woody cover	↓	↓[trend]	No effect
Woody species richness	↓	↓	No effect
Herbaceous biomass	No effect	No effect	No effect

### 4.1 Individual effects of herbivores on oak performance and forest communities

Extensive literature from North America and Europe clearly indicates that deer herbivory often limits the growth, abundance, and/or survival of young oaks in Mediterranean (Bartolome et al., 2002; Davis et al., 2011; Griffin, 1971; Long et al., 2007; Perrin, Kelly, & Mitchell, 2006; Tyler et al., 2008; White, 1966) and temperate systems (Bradshaw & Waller, 2016; Inouye, Allison, & Johnson, 1994; Kelly, 2002; Russell & Fowler, 2004; Stange & Shea, 1998). Results from our 14‐year‐old exclosure experiment support and expand upon these studies, showing that black‐tailed deer have been a critical factor mediating the growth, architecture, and survival of juvenile coast live oak. Exposure to deer herbivory accounted for nearly all of the tree mortality observed in this long‐term study. Deer also maintained surviving juvenile oaks at nearly the same height over the entire study, contrasting greatly with oaks protected from deer, which exhibited a 3.1‐fold increase in height during the same period (Figure 1). In addition, deer herbivory caused reductions in the stem diameter, leaf abundance, and canopy area of individual trees (Figure 2a–e). We hypothesize that these impacts collectively led to reductions in carbon reserves, which in turn lowered growth, as expressed in the reduced basal stem diameter of trees. Lastly, our results suggest that intense deer herbivory prevented vertical growth, but did not fully limit lateral growth, such that the shape of trees shifted over time, with juveniles becoming much wider than tall (Figure 2d). In summary, results from the 14‐year deer exclosure experiment showed that black‐tailed deer had large negative effects on juvenile coast live oaks—increasing their mortality and maintaining survivors in a stunted state aboveground that prevented them from reaching the sapling stage and becoming reproductive.

Results from our eight‐year factorial exclosure experiment at two sites provide an expanded but more complicated picture of the context‐dependence effects of mammalian consumers on oaks and the surrounding community. In contrast to our earlier experiment, in most cases black‐tailed deer by themselves did not have effects on oaks (Table 1). The primary exception to this pattern was that deer reduced the richness of woody species and the cover of nonoak woody taxa. We suggest that two factors may explain the overall differences in the effects of deer between our two experiments. First, we suspect that deer were more abundant—and herbivory on oaks more intense—in the heterogeneous savanna/woodland habitat where our 14‐year‐old experiment was located, compared to the closed‐canopy woodland habitat where our 8‐year‐old experiment was conducted. This is because the former environment likely offered a greater array of desirable forage species. Second, our 14‐year‐old experiment focused exclusively on individual oak trees whereas our 8‐year‐old experiment addressed the effects of herbivores on the entire plant community, including oaks and other woody understory species. Thus, the responses of oaks to deer in the second experiment may have been muted, or at least complicated, by the responses of their neighboring plants.

In contrast to the findings for deer, our 8‐year‐old experiment showed that small mammals had strong negative effects on the abundance and/or size of seedlings for three oak species, and these effects scaled up to the community level in the form of reductions in oak and nonoak woody cover and woody species richness (Table 1). The effects of small mammals on nonoak woody cover (largely poison oak, *Toxicodendron diversilobum*) were especially pronounced—with these consumers causing on average a 30% decrease in cover (Figure 6b). These strong effects were particularly impressively given that we were only able to reduce the abundance of small mammals, rather than completely exclude them. Our results, combined with those from other studies, clearly show that small mammals often reduce the survival and growth of oak seedlings and substantially deplete acorn crops (López‐Sánchez et al., 2019; MacDougall et al., 2010; McLaughlin & Zavaleta, 2013; Tyler et al., 2008).

Mammalian herbivores are well known to alter forest structure by affecting the cover and richness of woody species (Gill & Beardall, 2001; Rossell, Patch, & Salmons, 2007; Seabloom, Borer, Martin, & Orrock, 2009). Researchers have consistently found that deer reduce the cover of understory species, with areas experiencing high‐intensity browsing exhibiting reduced recruitment of woody taxa (Augustine & Jordan, 1998; deCalesta & Stout, 1997; Rooney, 2009). In contrast to woody cover, previous studies have documented that the effects of deer on woody species richness are often highly context dependent, ranging from positive to negative, with the direction thought to be dependent on site productivity and deer browsing pressure (Casabon & Pothier, 2008; Muñoz et al., 2009). Deer usually decrease species richness in low‐productivity sites and at high‐browsing pressures and increase species richness at high‐productivity sites and at moderate‐browsing pressure (Bakker et al., 2006; Royo, Collins, Adams, Kirschbaum, & Carson, 2010).

Numerous studies have shown that small mammals can have major effects on the characteristics of plant communities in grasslands and other more open, less tree‐dominated systems (e.g., Báez, Collins, Lightfoot, & Koontz, 2006; Bagchi, Namgail, & Ritchie, 2006; Bakker et al., 2006; Maron, Pearson, Potter, & Ortega, 2012; Olofsson, Hulme, Oksanen, & Suominen, 2004). In contrast, we are not aware of many studies that have documented community‐level effects of small mammals in forested ecosystems, and suspect that this possibility is infrequently considered. For instance, Ayres and Lombardero (2000) included only hares and large mammals on the list of mammalian herbivores that are significant agents of biological disturbance in North American forests. In our study, we have detected significant effects of small mammals on the cover and richness of woody understory species in forests, and we hypothesize that these abundant consumers have greater effects on forest communities than currently appreciated. As we have discussed previously, there is abundant evidence that small mammals have considerable negative effects on juvenile tree species in other systems and we suspect that such influences frequently scale up to result in community‐level consequences. Indeed, in a comprehensive study of factors limiting oak recruitment in North America, MacDougall et al. (2010) concluded that small mammals were a much more important factor than deer—and these smaller consumers are widely recognized to have effects on the structure and composition of forest communities.

### 4.2 Interactive herbivore effects on oak performance

In addition to the direct effects of deer and small mammals already discussed, we detected numerous interactions between these two consumer groups. As summarized in Table 1, we found that deer had a negative effect on the abundance of black oak and coast live oak juveniles only when small mammals were present, not when their abundance was reduced with fencing (Figure 3b,c). One possible explanation for this context‐dependent outcome is that small mammals greatly reduced nonoak woody cover (Figure 6b), which may have increased the accessibility and/or apparency of oak seedlings to browsing deer (sensu associational effects; Root, 1973, Hay, 1986). We also found that deer had a positive influence on the cover of understory oaks but only when small mammals were reduced with fencing. We hypothesize that deer had a positive influence on oak dominance because nonoak woody cover was much greater when small mammals were reduced (Figure 6b) and the cover reductions caused by deer browsing reduced the intensity of competition between juvenile oaks and neighboring, faster‐growing woody understory species.

Another form of interaction was detected for the abundance of white oak seedlings, although the effect was more complicated than the other examples. Seedling abundance was lowest when both herbivores were present and when both were experimentally excluded/reduced (Figure 3a). We hypothesize that the abundance of white oak seedling was low when both consumers were present because of intense herbivory and when both consumers were excluded/reduced because of intense competition with nonoak woody species, which were exceedingly dominant under these conditions, approaching 60% cover on average.

### 4.3 Recruitment under chronic browsing pressure

Given the intensity of deer herbivory on oaks in our 14‐year‐old experiment and in many other areas throughout California and beyond, it seems remarkable that any level of tree recruitment occurs. Indeed, understanding how juvenile oaks are able to escape this consumer pressure to reach the sapling stage is a critically important question in forest ecology and management. In our system, juvenile oaks that reach the sapling stage—and thus largely escape deer browsing—are a rarity but do occur periodically. In many cases, this occurs because juvenile oaks gain protection from chronic deer herbivory by either growing in association with physical structures, such as stonewalls, or established woody species. Carson, Banta, Royo, and Kirschbaum (2005) found similar results in the eastern United States where large boulders prevented deer access and thus served as refugia for five dominant tree species. Similarly, Grisez (1960) showed that deer browsing was lower and seedling density greater when woody species grew within large slash piles, presumably because deer were not afforded access to these areas. In addition, numerous studies have shown that growth, density, and/or recruitment of oaks is often greater within dense woody vegetation and berry thickets that exclude or greatly reduce deer browsing (Russell & Fowler, 2004; Williams, Westrick, & Williams, 2006). For example, numerous studies have shown that the recruitment and growth of juvenile oaks are greater within shrub canopies, at least in part due to reductions in the occurrence and/or intensity of mammalian herbivory (Callaway, 1992; Callaway & D’Antonio, 1991; Gómez‐Aparicio, Zamora, Castro, & Hódar, 2008; Perea, López‐Sánchez, & Dirzo, 2017). Ripple and Beschta (2008) also reported greater oak recruitment in refugia sites where physical barriers prevented deer access, such as strips of land between highways and rivers.

In our system, we also noted a third way that juvenile oaks were able to escape the consumer pressure from deer. When growing without physical or biotic protection, juvenile coast live oaks regularly developed “skirt” structures at their bases that reduced the accessibility of central shoots to browsing deer and thus allowed trees to transition from small bushy shrubs to more upright trees. Once they grew enough laterally, the bushy structure of juveniles provided sufficient protection so that a central stem could escape herbivory and subsequently bolt. Griffin (1971) reported this same phenomenon, noting that coast live oak frequently escaped deer browsing by developing low‐growing “hedges” that keep deer away from the central area, thereby allowing more rapid vertical growth of interior stems. In our system, we hypothesize that juvenile coast live oaks are unable to grow above the browse line to reach the sapling stage unless they associate with vegetation or abiotic structures that provide them protection from herbivory or themselves develop architectures (lateral “skirts” at their bases) that do so.

### 4.4 Conclusions

In conclusion, we have presented results from two long‐term exclosure experiments showing that deer and small mammals are important forces influencing oak populations and associated woody communities in northern California. Our work adds to a growing number of studies documenting that mammalian herbivores may play an important role in the observed declines in recruitment and regeneration in North American forests. We have shown that mammalian consumers play crucial roles in limiting oak recruitment by reducing seedling abundance, maintaining trees in stunted states, and preventing them from reaching sapling stages and becoming reproductive. Our work also reveals that interactions between large and small mammals can alter the intensity and direction of their effects, highlighting the importance of performing fully factorial experiments that examine the context‐dependent effects of multiple herbivores on tree recruitment. Finally, given that oak species are recognized as keystone species that provide critical resources and create habitat for many other species (MacDougall et al., 2010; Manning et al., 2006; Zavaleta et al., 2007), changes in oak recruitment could have wide‐ranging effects on other species that extend far beyond the three species considered in this study.

## CONFLICT OF INTEREST

All three authors certify that they do not have any conflict of interest to disclose.

## AUTHOR CONTRIBUTION

**J. Hall Cushman:** Conceptualization (lead); Formal analysis (equal); Funding acquisition (lead); Investigation (lead); Methodology (equal); Project administration (lead); Resources (lead); Supervision (lead); Visualization (equal); Writing‐original draft (lead); Writing‐review & editing (lead). **Laura E. Saunders:** Data curation (lead); Formal analysis (equal); Investigation (equal); Methodology (lead); Visualization (equal); Writing‐original draft (equal). **Tyler K. Refsland:** Formal analysis (equal); Visualization (equal); Writing‐original draft (supporting); Writing‐review & editing (supporting).

## Data Availability

Data are published on Dryad—https://doi.org/10.5061/dryad.nk98sf7r2.
